# Distinct expression patterns of HCN channels in HL-1 cardiomyocytes

**DOI:** 10.1186/s12860-015-0065-5

**Published:** 2015-07-04

**Authors:** Anne Günther, Arnd Baumann

**Affiliations:** Institute of Complex Systems, Cellular Biophysics (ICS-4), Wilhelm-Johnen-Straße, Forschungszentrum Jülich, D-52425 Jülich, Germany

**Keywords:** Pacemaker channel, Cardiac cells, Transcript quantification, Signaling, Contractile activity

## Abstract

**Background:**

Cardiac rhythmic activity is initiated in functionally specialized areas of the heart. Hyperpolarization-activated and cyclic nucleotide-gated (HCN) channels are fundamental for these processes of cardiac physiology.

**Results:**

Here we investigated transcript and protein expression patterns of HCN channels in HL-1 cardiomyocytes using a combination of quantitative PCR analysis and immunocytochemistry. Gene expression profiles of *hcn1*, *hcn2* and *hcn4* were acutely affected during HL-1 cell propagation. In addition, distinct expression patterns were uncovered for HCN1, HCN2 and HCN4 proteins.

**Conclusions:**

Our results suggest that HCN channel isoforms might be involved in the concerted differentiation of HL-1 cells and may indirectly affect the occurrence of contractile HL-1 cell activity. We expect that these findings will promote studies on other molecular markers that contribute to cardiac physiology.

**Electronic supplementary material:**

The online version of this article (doi:10.1186/s12860-015-0065-5) contains supplementary material, which is available to authorized users.

## Background

Cardiac autonomous activity originates in the sinoatrial node (SAN) where primary pacemaker cells generate spontaneous rhythmic action potentials that trigger heart muscle contraction. Generation of rhythmic action potentials in the heart involves several channel proteins including T- and L-type Ca^2+^ channels, a K^+^ channel and a pacemaker channel [1]. Activation of T- and L-type Ca^2+^ channels results in depolarization, followed by repolarization of the membrane potential due to opening of K^+^ channels. Near the K^+^ equilibrium potential pacemaker channels open and give way to an inward Na^+^ current. Subsequent membrane depolarization towards the threshold voltage induces a new action potential. This pacemaker current, also termed *I*_h_ or *I*_f_, is an essential component of cardiac automaticity [2, 3].

Hyperpolarization-activated and cyclic nucleotide-gated (HCN) channels are the molecular basis of *I*_f_ [4]. In mammals four HCN channel subunits (HCN1 – 4) with different biophysical properties and expression patterns have been identified [5, 6]. In the murine SAN the HCN4 isoform is the predominant subunit [7–9]. Although reports on expression of HCN1 – 4 in different species are not consistent, HCN1 and HCN2 have been identified consistently in murine cardiac tissue [7–10]. Transgenic studies in mice further emphasized the importance of HCN channels for cardiac function. Knockout of HCN4 as well as a point mutation in the cyclic nucleotide-binding site of HCN4 caused embryonic lethality due to cardiac malfunction [11, 12]. Conditional knockout of HCN4 in adult mice was either lethal [13] or induced cardiac arrhythmia with a reduction of *I*_f_ by about 75 % in the SAN [14]. Knockout of HCN2 reduced sinoatrial *I*_f_ by approximately 30 % [15], while a combined knockout of HCN2 and HCN4 caused a complete disruption of ventricular *I*_f_ [16].

However, little is known about the link between cardiac functionality and HCN channel isoform expression on the cellular level. Here we employed HL-1 cardiomyocytes, a murine atrial myocyte-based cell line that mimic properties of adult cardiomyocytes and express cardiac signature genes [17, 18]. Due to their ability to develop features similar to cardiac tissue, including spontaneous contractile activity [18], HL-1 cells are a versatile cellular model system for investigating molecular components of cardiac physiology *in vitro*.

In this study we focused on expression profiles of HCN channel isoforms in HL-1 cells. Differential expression of all four HCN isoform genes was examined via quantitative PCR. As an independent method, immunocytochemistry was used to analyze the presence of these proteins in HL-1 cells. Notably, HCN channel isoforms were not detected in contracting HL-1 cells. However, our results suggest a direct correlation between *hcn* gene expression and HL-1 cell differentiation. Thus HL-1 cells can serve as a model system for *in vitro* studies of cardiomyocyte development and differentiation.

## Methods

### HL-1 cells

HL-1 cells were obtained from Dr. W. C. Claycomb (Louisiana State University Health Science Center, New Orleans, LA, USA) and were cultured as recommended [17]. Briefly, cells were maintained in Claycomb Medium (SAFC Biosciences, Hamburg, Germany) supplemented with 10 % FBS (fetal bovine serum, SAFC Biosciences), 100 μM noradrenaline (Sigma-Aldrich, Munich, Germany), 2 mM L-glutamine (Life Technologies, Darmstadt, Germany), 300 μM ascorbic acid (Sigma-Aldrich), and 100U/ml:100 μg/ml streptomycin:penicillin (Life Technologies). Cells were cultivated on dishes coated with 0.02 % gelatin (Life Technologies) and 12.5 μg/ml fibronectin (from bovine plasma, Sigma-Aldrich) at 37 °C, 5 % CO_2_, and 95 % relative humidity. For imaging, cells were cultivated either on pre-coated glass coverslips or on 35 mm cell culture dishes with glass bottom (Ibidi, Martinsried, Germany).

### RNA preparation and cDNA synthesis

Total RNA was isolated from HL-1 cells and mouse brain using the DNA/RNA/Protein AllPrep® Kit (Qiagen, Hilden, Germany) according to the supplier's protocol. Animal experiments were performed in accordance with institutional protocols in compliance with national and international guidelines (Directive 2010/63/EU). RNA samples were split for two independent first-strand cDNA syntheses using Oligo-dT primers (Qiagen) and Moloney Murine Leukemia Virus reverse transcriptase (M-MLV-RT, Life Technologies) according to the supplier’s protocol.

### Quantification of gene expression by real-time PCR

Thermocycling was performed in a LightCycler 1.5 (Roche, Mannheim, Germany) using the QuantiTect SYBR Green PCR Kit (Qiagen) according to the supplier’s protocol. Gene-specific primers were purchased from MWG Operon (Ebersberg, Germany). Specificity and efficiency of primers (Table 1) were confirmed via BLAST analysis and PCR on cloned gene fragments. qPCR reactions were performed on 1 μl aliquots of first-strand cDNA samples in a total volume of 20 μl. The *gapdh* primers were designed to bind in exons separated by an intron of 134 bp to check for genomic impurities. qPCR runs were concluded by generating a melting curve to confirm homogeneity of amplified fragments. Results were analyzed using the C_t_ method. Gene expression levels were normalized to the housekeeping gene *gapdh*. Data sets generated in the course of one experimental series were normalized to values at a specified culture density (*i. e.* approximately 50 % confluency). Samples were grouped according to visually determined culture densities: five groups were defined, *i. e.* with 1–20 %, 21–40 %, 41–60 %, 61–80 %, and 81–100 % cell density. For analysis, mean ± s.e.m. values were calculated.

**Table 1:** Primer pairs for qPCR on HL-1 cell and mouse brain cDNA

Protein	Gene	Primer	Sequence (5‘→3‘)	T_*m*_	Amplicon size
GAPDH	*gapdh*	Forward	GGTATCGTGGAAGGACTCATG	62 °C	150 bp/284 bp
		Reverse	GCTGCCAAGGCTGTGGGC		
HCN1	*hcn1*	Forward	ACTGTGGGCGAATCCCTGG	62 °C	184 bp
		Reverse	CCACCAGCAGCTGTGCAGA		
HCN2	*hcn2*	Forward	GGAGAATGCCATCATCCAGG	62 °C	149 bp
		Reverse	CAGCAGGCTGTGGCCATGA		
HCN3	*hcn3*	Forward	TGGGAACCACTGGTGCACG	62 °C	141 bp
		Reverse	TGAGCGTCTAGCAGATCGAG		
HCN4	*hcn4*	Forward	CACGACCTCAACTCAGGCG	62 °C	153 bp
		Reverse	CAGCGGGGTCCATATAACAG		

Primer sequences and amplicon sizes are based on mouse sequences (accession numbers: NM_008084.2 (*gapdh*), NM_010408.3 (*hcn1*), NM_008226.2 (*hcn2*), NM_008227.1 (*hcn3*), and NM_001081192.1 (*hcn4*)). Amplicon sizes of *gapdh* fragments are indicated for cDNA (150 bp) and genomic DNA (284 bp).

### Statistical analysis

All data are represented as mean ± s.e.m. (standard error of the mean). The two-tailed independent Student’s *t* test was applied for calculation of *p* values. One-way ANOVA (analysis of variance) was performed using GraphPad Prism v.5.04 for Windows (GraphPad Prism Software, San Diego, California, USA) for analysis of gene expression profiles. A *p* value of <0.05 was considered significant.

### Antibodies

Primary antibodies for immunocytochemistry were anti-mHCN1 (HCN1α, guinea pig, 1:500), anti-mHCN2 (HCN2α, rabbit, 1:500), and anti-mHCN4 (PG2-1A4, rat, 1:2) (Additional file 1: Table S1). Secondary antibodies were anti-rabbit-Cy2 (polyclonal, 1:400, Dianova), anti-rat-Cy3 (polyclonal, 1:500, Dianova), and anti-guinea pig-A594 (polyclonal, 1:500, Dianova).

### Immunocytochemistry

For immunostaining, cells were fixed for 10 min in 4 % (w/v) paraformaldehyde. After washing with PBS (130 mM NaCl, 70 mM Na_2_HPO_4_, 30 mM NaH_2_PO_4_, pH 7.4), unspecific binding of antibodies was blocked for 30 min in CT (5 % (v/v) chemiblocker (Chemicon, Darmstadt, Germany) and 0.5 % (v/v) Triton-X in PBS). Incubation with primary antibodies was performed for 60 min in CT. Secondary antibodies in CT were added for 60 min after washing with PBS. Samples were mounted on microscope slides with Aqua-Poly/Mount (Polysciences, Eppelheim, Germany) or preserved in PBS.

### Microscopy

Fluorescent images were obtained with an inverted confocal microscope (TCS SP5II, Leica, Wetzlar, Germany) using a 63x oil immersion objective. Z-stacks (5 x 1 μm) were registered, and maximum intensity projections were generated using WCIF ImageJ 1.37c software. For identification of contracting cells, 15 sequential pictures (3frames/s) were taken using an inverted microscope (Axio Vert.A1, Zeiss, Göttingen, Germany) equipped with a digital SLR camera (Nikon D200, Nikon, Düsseldorf, Germany). Pictures were converted into video clips (Additional file 2: Video S1) (3frames/s) using ImageJ software.

## Results

Cardiac contraction strongly depends on consecutive activity of different voltage-dependent ion channel classes [1]. In the heart, HCN channels not only restore the resting membrane potential from hyperpolarized values but also contribute to a novel depolarization due to their pronounced Na^+^ conductance. In this study we examined expression patterns of four HCN channel isoforms in HL-1 cells, both on the transcript and on the protein level.

### Gene expression of HCN channel isoforms in HL-1 cells

**Fig. 1:**
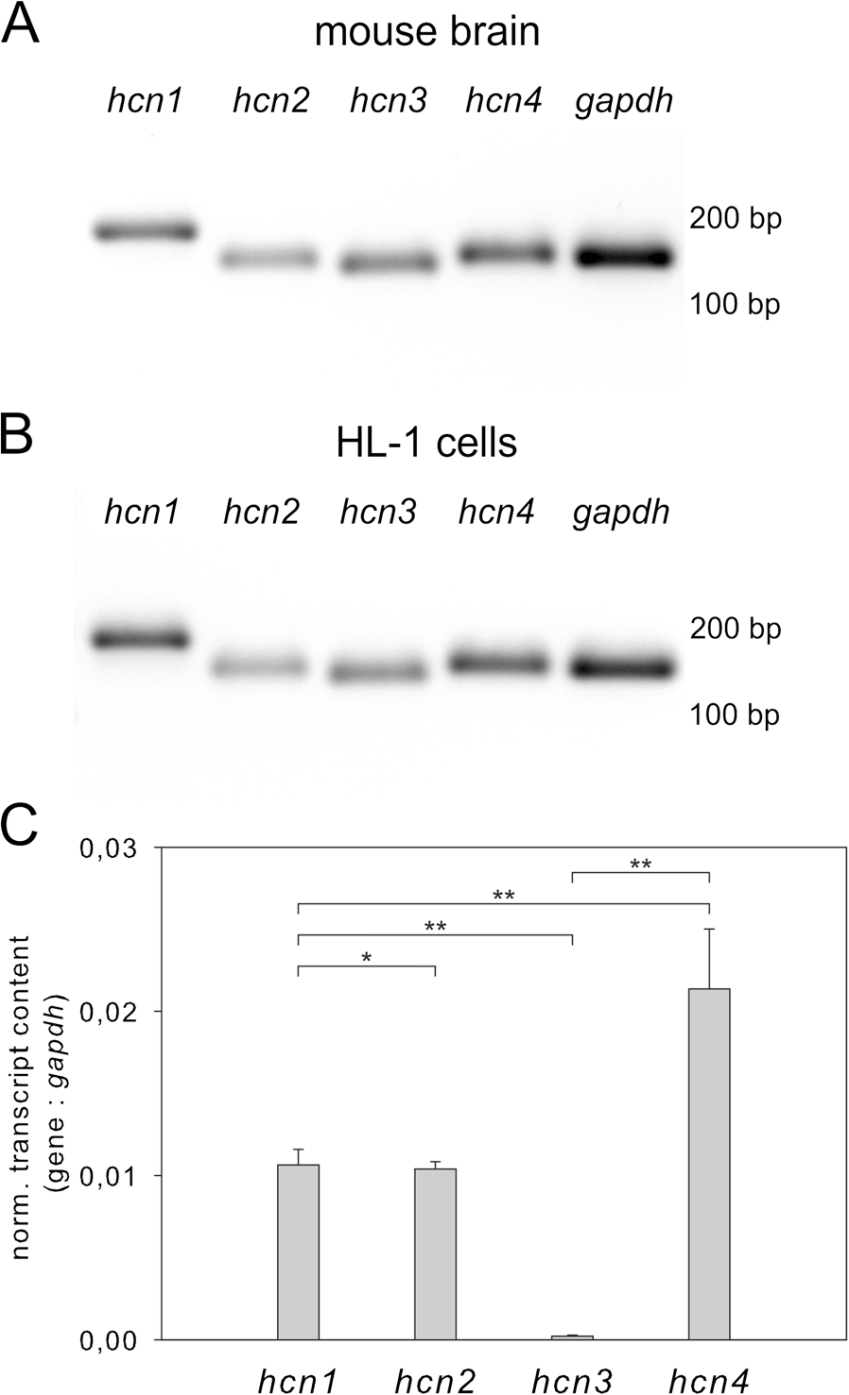
Gene expression of HCN channel isoforms in mouse brain and HL-1 cells. PCR analysis of *hcn1 – 4* and *gapdh* gene expression on mouse brain cDNA (**a**) and HL-1 cell cDNA (**b**). Sizes of marker bands are indicated on the right. Gene expression levels were determined via qPCR on cDNA of confluent HL-1 cell cultures. Transcript expression levels for *hcn 1 – 4* genes normalized to *gapdh* are shown in (**c**). Data points depict mean ± s.e.m. (*n* = 4) and Student's *t* test was performed (*p < 0,9; **p < 0,005)

For investigation of HCN channel isoform gene expression, specific primers were designed to amplify defined *hcn* fragments. The specificity of these primers was confirmed on mouse brain cDNA (Fig. 1a), where *hcn1 - 4* isoforms are known to be expressed [5, 6], as well as on HL-1 cell-derived cDNA (Fig. 1b). Amplified fragments displayed expected sizes, *i. e.* 184 bp for *hcn1*, 149 bp for *hcn2*, 141 bp for *hcn3*, 153 bp for *hcn4*, and 150 bp for *gapdh*. Gene expression of *hcn1 – 4* in confluent HL-1 cell culture was determined via qPCR and normalized to *gapdh* (Fig. 1c). While transcript levels of *hcn1* and *hcn2* were rather similar, *hcn4* transcripts were almost twice as abundant as *hcn1* or *hcn2*. In this quantitative analysis only marginal amounts of *hcn3* transcripts were detected.

HL-1 cells develop contractile activity at high culture densities, implicating cell confluency as an important factor of HL-1 cell differentiation. To examine whether the development of contractile HL-1 cell activity correlates with *hcn* gene expression, transcript levels of *hcn1*, *hcn2* and *hcn4* were quantified at different HL-1 culture densities. The following cultivation strategies were applied: (i) cells were plated at different densities, and mRNA was isolated after 4d in culture; (ii) cells were plated at defined densities, and mRNA was isolated after different cultivation times (1d–4d after plating). Samples were grouped according to their respective culture densities that were visually determined prior to mRNA isolation. Transcript expression was examined via qPCR and normalized to the housekeeping gene *gapdh*.

**Fig. 2:**
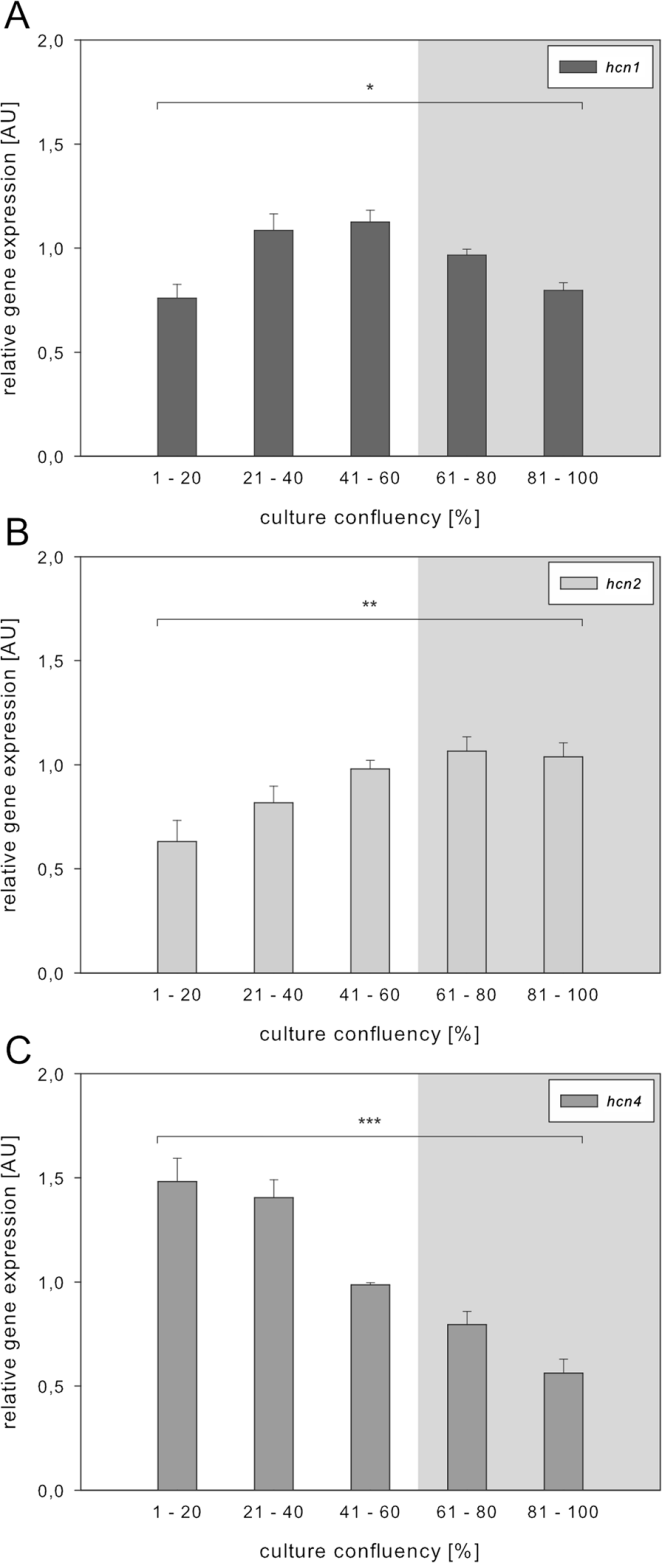
Expression of *hcn* genes in HL-1 cell samples obtained at different culture densities. Gene expression levels were determined via qPCR on cDNA from HL-1 cells propagated at different culture densities according to cultivation strategy (i). Quantification of genes (y-axis) is shown in relation to culture confluency (x-axis). Values for *hcn1* (**a**), *hcn2* (**b**), and *hcn4* (**c**) were normalized to *gapdh* (see *2.3.*). Samples were grouped according to visually determined culture densities. Grey inlays denote conditions where HL-1 cells displayed strong contractile activity. Data points depict mean ± s.e.m. and one-way ANOVA was performed for each group (for culture confluency: *F_(4,27)_ = 8.298, *p* < 0.0002; **F_(4,28)_ = 5.708, *p* < 0.002; ***F_(4,25)_ = 31.22, *p* < 0.0001)

Striking gene-specific changes in expression profiles were observed (Fig. 2). Expression of *hcn1* and *hcn2* increased with increasing culture density. For both genes the highest transcript levels were measured at about 60 % cell confluency. Notably, in cultures reaching 100 % confluency *hcn1* expression decreased almost to the starting level (Fig. 2a), whereas *hcn2* levels remained elevated (Fig. 2b). In contrast to *hcn1* and *hcn2*, *hcn4* expression was high at low cell confluency. With increasing culture density, however, *hcn4* expression decreased almost linearly (Fig. 2c). Gene expression profiles were similar when comparing data sets generated according to cultivation strategy (i) or (ii) (Additional file 1: Figure S1). Based on these transcript expression profiles one might speculate that individual *hcn* isoforms play a role in HL-1 differentiation as well as in establishing HL-1 cell contractile activity. However, qPCR data was obtained from the entire cell culture samples not providing information on the individual cell level. Therefore, we examined the distribution of HCN proteins via immunocytochemistry.

### Analysis of HCN channel isoform expression in HL-1 cells

**Fig. 3:**
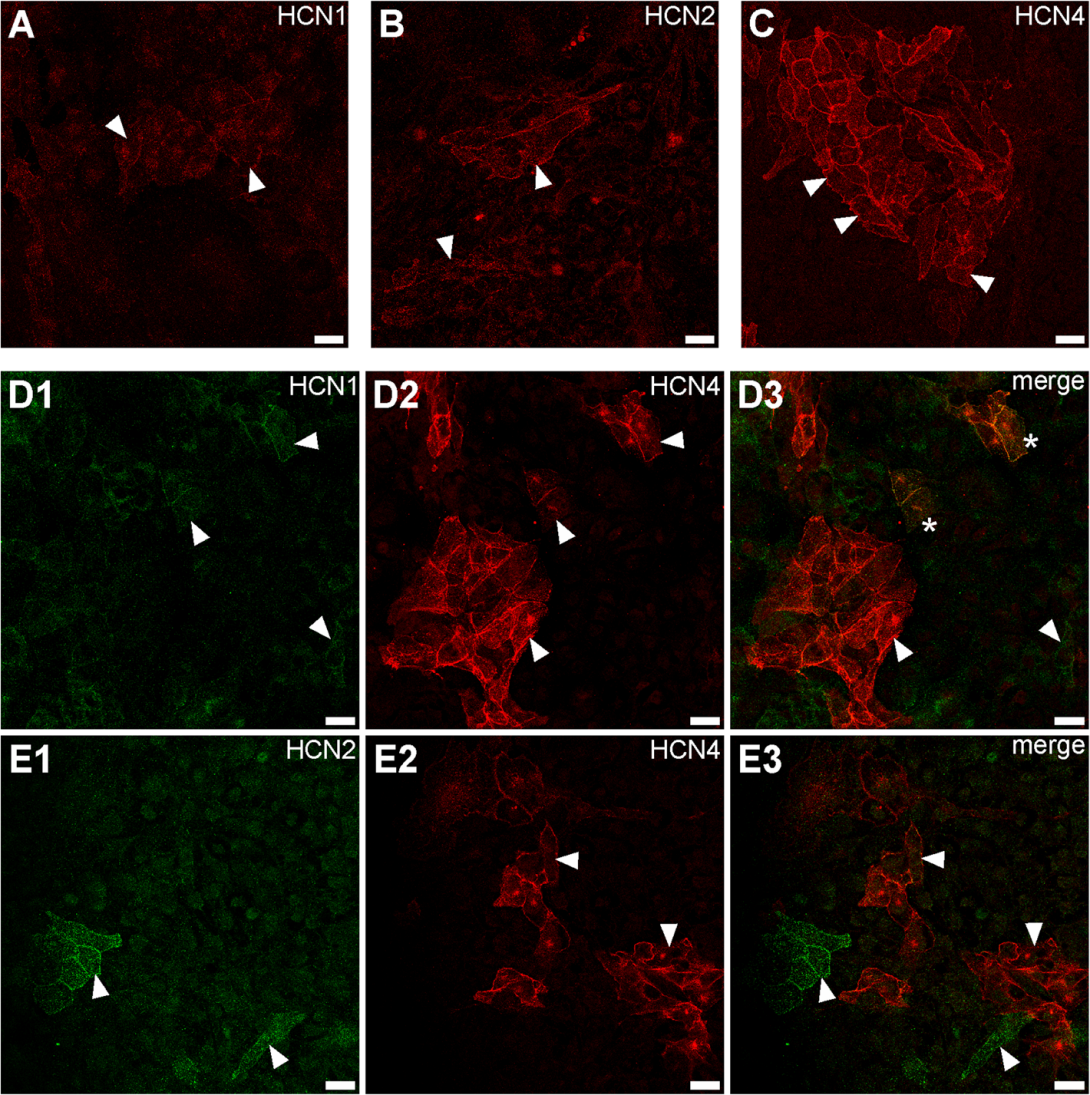
Expression of HCN channel isoforms in HL-1 cells. Immunostaining of HCN1 (**a**), HCN2 (**b**), and HCN4 (**c**) in confluent HL-1 cell cultures was performed with HCN isoform-specific primary antibodies (see Additional file 1: Table S1) followed by fluorescently labeled secondary antibodies. HL-1 cells were double-immunostained for HCN1 (d1) and HCN4 (d2) and for HCN2 (e1) and HCN4 (e2); merged images of double-immunostainings are shown in (d3) and (e3), respectively. Some cells specifically stained for either isoform are indicated by arrowheads; co-immunolabeled cells are marked by asterisks. Scale bars specify 20 μm

In order to gain insight into expression patterns of HCN proteins in individual HL-1 cells we performed a series of immunocytochemical stainings with HCN isoform-specific primary antibodies. Expression of HCN1, HCN2 and HCN4 was reliably detected (Fig. 3a–3c), whereas detection of HCN3 proteins failed. HCN1, HCN2 and HCN4 were primarily located at the plasma membrane (Fig. 3a–3c, arrowheads). Notably, only a fraction of HL-1 cells in culture expressed HCN channel proteins: We observed approximately 3 % HCN1-positive, 1 % HCN2-positive, and 10 % HCN4-positive cells in confluent HL-1 cell cultures (Additional file 1: Figure S2). HCN1 expression was detected in single cells as well as in small cell clusters. Expression of HCN2 was found mostly in single cells. In contrast, HCN4-expressing cells tended to form large cell clusters. Co-immunostaining revealed co-expression of HCN1 and HCN4 in approximately 0.5 % of cells (Fig. 3d3, asterisks), however, the majority of HCN1 and HCN4-positive cells expressed only one HCN isoform (Fig. 3d3, arrowheads). Co-immunolabeling of HCN2 and HCN4 showed that these proteins were expressed in different cells or cell clusters (Fig. 3e3). Similar expression patterns have been reported *in vivo* for these HCN isoforms [10, 19]. Our findings on cell specific and restricted expression of HCN channel proteins suggest that HL-1 cells in culture occur in various states, rather than being a homogeneous population of cells.

### HCN isoform expression and cell contraction

**Figure 4:**
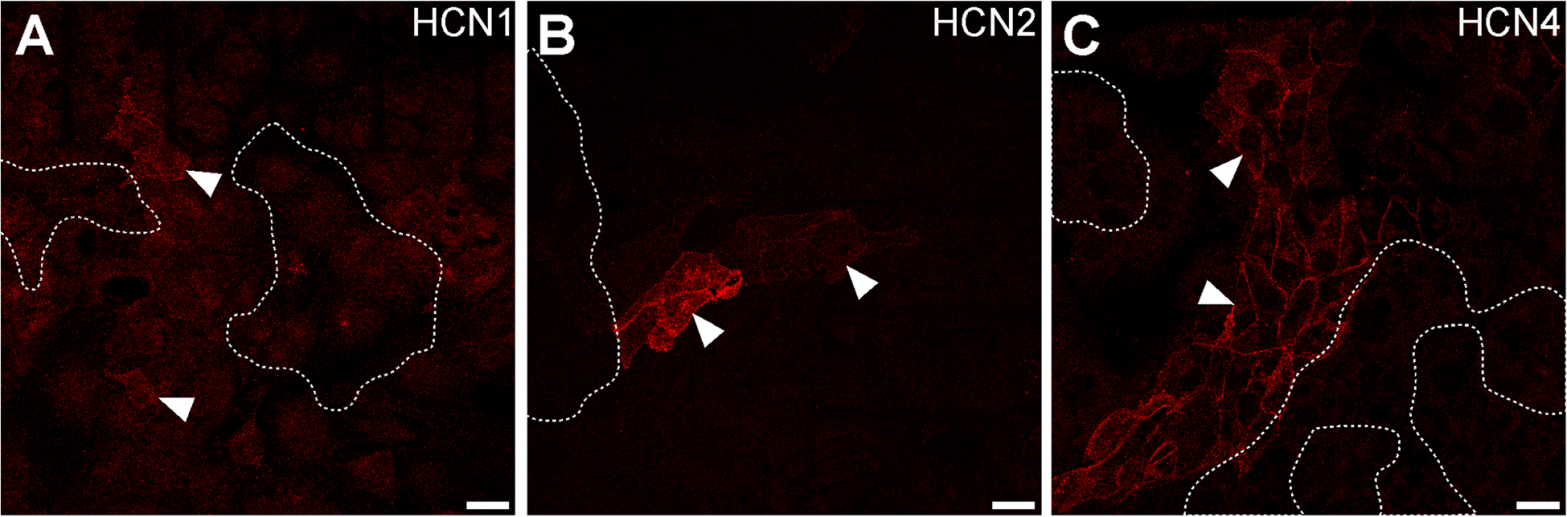
Expression of HCN isoforms in HL-1 cell cultures displaying contractile activity. Contractile activity in confluent HL-1 cell cultures was documented (dotted lines) before samples were immunostained with specific primary antibodies (see Additional file 1: Table S1) and fluorescently labeled secondary antibodies. Images of HL-1 cell contraction and immunostainings were superimposed based on underlying grids (see Additional file 1: Figure S3). Areas of contractile cell clusters are indicated by dotted lines. Immunolabeling of HCN1 (**a**), HCN2 (**b**), and HCN4 (**c**) is depicted. Arrowheads indicate some cells specifically stained for HCN isoforms. Scale bars specify 20 μm

To investigate whether contractile HL-1 cell activity can be attributed to the expression of HCN isoforms, spontaneously contractile cell clusters were microscopically identified on coverslips with integrated grid patterns. Subsequently, samples were fixed and stained for HCN isoforms. Images taken from live documentation and immunolabeling were superimposed and aligned based on the underlying grids (Additional file 1: Figure S3). At high culture densities HL-1 cells tend to grow in overlapping layers instead of a confluent monolayer. Superimposing live documentation images and maximal intensity projections occasionally suggested an overlap of HCN-expressing cells and spontaneously contracting areas (Additional file 1: Figure S3). However, this overlay was completely due to the layered growth of HL-1 cell cultures, since spontaneously contracting cells did not express any of the investigated proteins (Fig. 4). Nevertheless, cell clusters displaying strong contractile activity were often found in the vicinity of large HCN4-expressing cell clusters (Fig. 4c, Additional file 1: Figure S3).

During propagation, HL-1 cells acquire strikingly different morphologies. Heterogeneity is reflected both, on the phenotypic level, *e. g.,* contractile and non-contractile cells, as well as on the molecular level as demonstrated by the highly diverse expression patterns of HCN channels.

## Discussion

In recent years, knowledge of the molecular components that are instrumental in cardiac physiology has expanded significantly. Contribution of HCN channels to cardiac rhythmicity is well established and has been corroborated by several knockout studies [13, 14, 16]. However, only a few studies addressed the potential link between cardiac rhythmic activity and the expression of HCN channel isoforms on the cellular level. Here we studied an *in vitro* cell system of cardiac origin, the HL-1 cell line, to examine their molecular repertoire of HCN channels.

The vertebrate heart is separated into distinct, functionally specialized segments that are essential for cardiac rhythmicity. Expression of proteins in a developmentally specified manner is essential for cardiac function. Since analysis of cardiac processes *in vivo* is limited, a number of studies have been initiated in cellular model systems, such as cardiomyocytes or cardiac cells derived from murine embryonic stem cells (mESC) [20–22]. HL-1 cells are another versatile model for investigation of cardiac processes *in vitro*, due to their unique feature of retaining properties reminiscent of differentiated adult cardiomyocytes, like contractile activity, even during serial passaging [17]. Furthermore, HL-1 cells have been shown to express several cardiac marker genes, *e. g.,* HCN channel isoforms [18, 23].

In this study, differentiation of HL-1 cells under different cultivation conditions was assessed via the emergence of spontaneous contractile activity. We noticed a marked increase of *hcn1* and *hcn2* gene expression with rising culture density, that ceased with the appearance of strong contractile activity in the culture. In contrast, we observed that with increasing culture confluency, the amount of *hcn4* transcripts decreased to approximately 40 % of the transcript level expressed at low confluency (see Fig. 3). In native cardiac tissue, *hcn4* gene expression declines with increasing age of mice [24], and the functional contribution of HCN4 to cardiac rhythmicity *in vivo* is successively reduced during the animal's development [12–14].

Gene expression profiles can provide important information on constitutive or differential transcriptional activity in tissues. However, they do not provide conclusive information on the corresponding protein expression patterns. This has been emphasized previously for HCN channel expression in the heart, where transcript and protein expression levels are not consistent [25]. Similar results were observed in this study (Additional file 1: Figure S2). In HL-1 cells, HCN4 was the predominant isoform, followed by HCN1, whereas HCN2 was the least expressed. Remarkably, these results are analogous to reported expression patterns of HCN isoforms in the murine cardiac conduction system [10]. Furthermore, the property of HCN4-positive cells to form cell clusters has also been found at the center of the rabbit SAN [26], as well as in mESC-derived pacemaker cell clusters, expressing either HCN1 or HCN4 [27].

HCN isoforms are expressed in a highly differential manner in the heart that correlates with cardiac physiology [8–10, 19]. Designated expression is emphasized as an essential part of functional tissue differentiation as properties of HCN channels largely depend on subunit composition [28, 29]. Co-assembly of different HCN isoforms in heterologous expression systems as well as in cardiomyocytes, results in functional HCN channels with biophysical properties that differ from homomeric channels [28, 29]. However, detailed studies addressing HCN isoform co-assembly in the heart are lacking. In this study, we observed co-expression of HCN1 and HCN4 in some HL-1 cells, similar to reports in mESC-derived pacemaker cells [27]. Although we did not perform electrophysiological measurements, it can be hypothesized that co-assembly of HCN1 and HCN4 increases the functional repertoire of pacemaking channels in HL-1 cells.

Even though HCN channels have been attributed distinct functions in cardiac rhythmicity, little is known about these aspects on the cellular level. Expression of the HCN channel-based *I*_f_ current has been shown in mESC-derived contracting cells, where pharmacological blocking of *I*_f_ caused a reduction of the contraction frequency [30]. However, reports on HCN isoform expression in mESC-derived contracting cells are not consistent [22, 27, 30]. We found that contractile HL-1 cell clusters were devoid of HCN proteins, but cell clusters expressing HCN4 were frequently located in the vicinity of contracting cells. Thus, the endogenous presence of HCN channels seems not to be required for HL-1 cells to develop contractile activity. Nevertheless, HCN channel expression in neighboring cell clusters might contribute to signaling events that culminate in cell contraction of nearby clusters. This hypothesis is supported by recent findings showing that contractility of mESC-derived cell clusters relies on the expression of specific transcription factors, *e. g.,* Shox2 and GATA6, in adjacent, non-contractile cell clusters [20–22]. Notably, in mESC-derived model systems Shox2-positive cell clusters express HCN4 [21], and cGATA6/mink-positive cells possess *I*_f_ currents [22].

## Conclusions

In this study, the expression patterns of HCN channels were resolved in HL-1 cells, both on the transcript and on the protein level. Notably, only a small fraction of cells in a confluent culture express these channels suggesting that HL-1 cells are present in a variety of molecular and functional states. Whether HL-1 cell contraction relies on HCN channel expression in adjacent cells and/or which additional factors contribute to the generation of contractile activity, remains to be addressed. It will also be interesting to examine whether a direct correlation between HCN channel expression and differentiation into distinct cellular phenotypes can be revealed.

## Additional files

Additional file 1: Table S1. Primary antibodies for immunocytochemistry. **Figure S1**. Expression of *hcn* genes in samples obtained at different cell culture densities. **Figure S2**. Relative expression of HCN isoform transcript and protein in confluent HL-1 cell cultures. **Figure S3**. Expression of HCN4 in cultures displaying contractile activity. Additional file 2:
**Video S1. Video of HL-1 cells displaying contractile activity in confluent culture.**

## Abbreviations

ESC: Embryonic stem cell
*I*_f_: Funny current
HCN: Hyperpolarization-activated and cyclic-nucleotide gated
SAN: Sinoatrial node

## References

[1] Kaupp UB, Seifert R (2001). Molecular diversity of pacemaker ion channels. Annu Rev Physiol.

[2] Brown H, DiFrancesco D (1980). Voltage-clamp investigations of membrane currents underlying pace-maker activity in rabbit sino-atrial node. J Physiol.

[3] DiFrancesco D (1993). Pacemaker mechanisms in cardiac tissue. Annu Rev Physiol.

[4] Biel M, Wahl-Schott C, Michalakis S, Zong X (2009). Hyperpolarization-activated cation channels: from genes to function. Physiol Rev.

[5] Ludwig A, Zong X, Jeglitsch M, Hofmann F, Biel M (1998). A family of hyperpolarization-activated mammalian cation channels. Nature.

[6] Santoro B, Liu DT, Yao H, Bartsch D, Kandel ER, Siegelbaum SA, Tibbs GR (1998). Identification of a gene encoding a hyperpolarization-activated pacemaker channel of brain. Cell.

[7] Liu J, Noble PJ, Xiao G, Abdelrahman M, Dobrzynski H, Boyett MR, Lei M, Noble D (2008). Role of pacemaking current in cardiac nodes: insights from a comparative study of sinoatrial node and atrioventricular node. Prog Biophys Mol Biol.

[8] Marionneau C, Couette B, Liu J, Li H, Mangoni ME, Nargeot J, Lei M, Escande D, Demolombe S (2005). Specific pattern of ionic channel gene expression associated with pacemaker activity in the mouse heart. J Physiol.

[9] Moosmang S, Stieber J, Zong X, Biel M, Hofmann F, Ludwig A (2001). Cellular expression and functional characterization of four hyperpolarization-activated pacemaker channels in cardiac and neuronal tissues. Eur J Biochem FEBS.

[10] Herrmann S, Layh B, Ludwig A (2011). Novel insights into the distribution of cardiac HCN channels: an expression study in the mouse heart. J Mol Cell Cardiol.

[11] Harzheim D, Pfeiffer KH, Fabritz L, Kremmer E, Buch T, Waisman A, Kirchhof P, Kaupp UB, Seifert R (2008). Cardiac pacemaker function of HCN4 channels in mice is confined to embryonic development and requires cyclic AMP. EMBO J.

[12] Stieber J, Herrmann S, Feil S, Löster J, Feil R, Biel M, Hofmann F, Ludwig A (2003). The hyperpolarization-activated channel HCN4 is required for the generation of pacemaker action potentials in the embryonic heart. Proc Natl Acad Sci U S A.

[13] Baruscotti M, Bucchi A, Viscomi C, Mandelli G, Consalez G, Gnecchi-Rusconi T, Montano N, Casali KR, Micheloni S, Barbuti A, DiFrancesco D (2011). Deep bradycardia and heart block caused by inducible cardiac-specific knockout of the pacemaker channel gene Hcn4. Proc Natl Acad Sci U S A.

[14] Herrmann S, Stieber J, Stöckl G, Hofmann F, Ludwig A (2007). HCN4 provides a “depolarization reserve” and is not required for heart rate acceleration in mice. EMBO J.

[15] Ludwig A, Budde T, Stieber J, Moosmang S, Wahl C, Holthoff K, Langebartels A, Wotjak C, Munsch T, Zong X, Feil S, Feil R, Lancel M, Chien KR, Konnerth A, Pape H-C, Biel M, Hofmann F (2003). Absence epilepsy and sinus dysrhythmia in mice lacking the pacemaker channel HCN2. EMBO J.

[16] Hofmann F, Fabritz L, Stieber J, Schmitt J, Kirchhof P, Ludwig A, Herrmann S (2012). Ventricular HCN channels decrease the repolarization reserve in the hypertrophic heart. Cardiovasc Res.

[17] Claycomb WC, Lanson NA, Stallworth BS, Egeland DB, Delcarpio JB, Bahinski A, Izzo NJ (1998). HL-1 cells: a cardiac muscle cell line that contracts and retains phenotypic characteristics of the adult cardiomyocyte. Proc Natl Acad Sci U S A.

[18] White SM, Constantin PE, Claycomb WC (2004). Cardiac physiology at the cellular level: use of cultured HL-1 cardiomyocytes for studies of cardiac muscle cell structure and function. Am J Physiol Heart Circ Physiol.

[19] Liu J, Dobrzynski H, Yanni J, Boyett MR, Lei M (2007). Organisation of the mouse sinoatrial node: structure and expression of HCN channels. Cardiovasc Res.

[20] Hashem SI, Claycomb WC (2013). Genetic isolation of stem cell-derived pacemaker-nodal cardiac myocytes. Mol Cell Biochem.

[21] Hashem SI, Lam ML, Mihardja SS, White SM, Lee RJ, Claycomb WC (2013). Shox2 regulates the pacemaker gene program in embryoid bodies. Stem Cells Dev.

[22] White SM, Claycomb WC (2005). Embryonic stem cells form an organized, functional cardiac conduction system in vitro. Am J Physiol Heart Circ Physiol.

[23] Sartiani L, Bochet P, Cerbai E, Mugelli A, Fischmeister R (2002). Functional expression of the hyperpolarization-activated, non-selective cation current If in immortalized HL-1 cardiomyocytes. J Physiol.

[24] Yasui K, Liu W, Opthof T, Kada K, Lee JK, Kamiya K, Kodama I (2001). I(f) current and spontaneous activity in mouse embryonic ventricular myocytes. Circ Res.

[25] Calejo AI, Reverendo M, Silva VS, Pereira PM, Santos MAS, Zorec R, Gonçalves PP. Differences in the expression pattern of HCN isoforms among mammalian tissues: sources and implications. Mol Biol Rep. 2014;41:297–307. 10.1007/s11033-013-2862-2 24234751

[26] Brioschi C, Micheloni S, Tellez JO, Pisoni G, Longhi R, Moroni P, Billeter R, Barbuti A, Dobrzynski H, Boyett MR, DiFrancesco D, Baruscotti M (2009). Distribution of the pacemaker HCN4 channel mRNA and protein in the rabbit sinoatrial node. J Mol Cell Cardiol.

[27] Barbuti A, Crespi A, Capilupo D, Mazzocchi N, Baruscotti M, DiFrancesco D (2009). Molecular composition and functional properties of f-channels in murine embryonic stem cell-derived pacemaker cells. J Mol Cell Cardiol.

[28] Whitaker GM, Angoli D, Nazzari H, Shigemoto R, Accili EA (2007). HCN2 and HCN4 isoforms self-assemble and co-assemble with equal preference to form functional pacemaker channels. J Biol Chem.

[29] Zhang Q, Huang A, Lin Y-C, Yu H-G (2009). Associated changes in HCN2 and HCN4 transcripts and I(f) pacemaker current in myocytes. Biochim Biophys Acta.

[30] Qu Y, Whitaker GM, Hove-Madsen L, Tibbits GF, Accili EA (2008). Hyperpolarization-activated cyclic nucleotide-modulated “HCN” channels confer regular and faster rhythmicity to beating mouse embryonic stem cells. J Physiol.

