# Supplementary material for: Distinct expression patterns of HCN channels in HL-1 cardiomyocytes
Source: BMC Cell Biol. 2015 Jul 4;16:18. doi: 10.1186/s12860-015-0065-5 (PMC4490601; doi:10.1186/s12860-015-0065-5)
Supplement: Additional file 1: Table S1. — Primary antibodies for immunocytochemistry. Figure S1. Expression of hcn genes in samples obtained at different cell culture densities. Figure S2. Relative expression of HCN isoform transcript and protein in confluent HL-1 cell cultures. Figure S3. Expression of HCN4 in cultures displaying contractile activity. [file 12860_2015_65_MOESM1_ESM.docx]

**Table S1** Primary antibodies for immunocytochemistry

Names of primary antibodies and their characteristics are listed. The antigens used for immunization, *i.e.* amino acid (aa) positions in the respective HCN protein, are specified. [gp] guinea pig; [rb] rabbit; [rt] rat; [ms] mouse.

| **Antibody** | **type** | **host** | **antigen** | **construct** | **reference** |
| --- | --- | --- | --- | --- | --- |
| **HCN1 (HCN1α)** | polyclonal | gp | aa 1 – 18 (ms) | fusion protein | [1] |
| **HCN2 (HCN2α)** | polyclonal | rb | aa 798 – 863 (ms) | fusion protein | [1] |
| **HCN4 (PG2-1A4)** | monoclonal | rt | aa 1116 – 1201 (ms) | fusion protein | [1, 2] |

1. Fried H-U, Kaupp UB, Müller F: Hyperpolarization-activated and cyclic nucleotide-gated channels are differentially expressed in juxtaglomerular cells in the olfactory bulb of mice. *Cell Tissue Res* 2010, 339:463–479.

2. Mataruga A, Kremmer E, Müller F: Type 3a and type 3b OFF cone bipolar cells provide for the alternative rod pathway in the mouse retina. *J Comp Neurol* 2007, 502:1123–1137.

**

**

**Figure S1** Expression of *hcn* genes in samples obtained at different cell culture densities.

HL-1 cells were propagated according to either of the following cultivation strategies: (i) cells were plated at different densities, and mRNA was isolated after 4d in culture (A - C; n=6; depicted graphs are taken from Figure 2 for comparison); (ii) cells were plated at defined densities, and mRNA was isolated after different cultivation times (D - F; n=4). Gene expression levels were determined via qPCR. Quantification of genes (y-axis) is shown in relation to culture confluency (x-axis). Values for *hcn1* (A, D), *hcn2* (B, E), and *hcn4* (C, F) were normalized to *gapdh*. Samples were grouped according to visually determined culture densities. Data points depict mean ± s.e.m. and one-way ANOVA was performed for each group (for culture confluency: [*] F_(4,27)_=8.298, *p*<0.0002; [**] F_(4,28)_=5.708, *p*<0.002; [***] F_(4,25)_=31.22, *p*<0.0001; [** **] F_(3,17)_=13.31, *p*<0,0001; [** * **] F_(3,17)_=11.93, *p*<0,0002; [*** ***] F_(3,16)_=9.011, *p*<0.001). Unfortunately, we did not detect amplification of products in samples from 61 - 80% confluency prepared according to strategy (ii). Grey inlays denote conditions where HL-1 cells displayed strong contractile activity.

**

**

**Figure S2** Relative expression of HCN isoforms in confluent HL-1 cell cultures.

Gene expression levels were determined via qPCR on cDNA samples obtained from confluent HL-1 cell cultures. The relative amount [%] of transcripts for each *hcn* gene was calculated to the total amount of *hcn* transcripts (= 100%) in a sample (depicted graph for transcript values was taken from Figure 1 for comparison). The percentage of cells expressing either protein [%] was calculated to the total number of cells expressing HCN proteins (= 100%). Data depict mean ± s.e.m. (n=4) and Student's *t* test was performed (* *p*<0,00006; ** *p*<0,002).

**
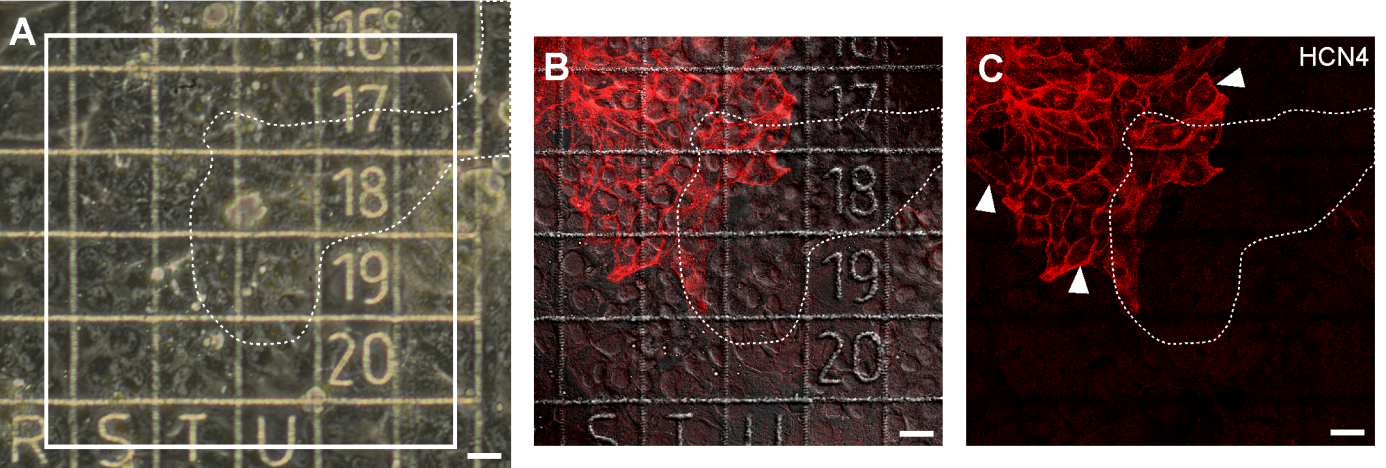
**

**Figure S3** Expression of HCN4 in cultures displaying contractile activity.

Cell contraction in confluent HL-1 cell cultures was documented microscopically before immunostaining was performed. Cell clusters showing strong contractile activity were identified (dotted lines) based on video clips of live documentation (see Video S1). A single frame of live cell documentation is shown in (A); the white box denotes the area shown in (B) and (C). Images of HL-1 cell contraction and immunostaining were superimposed according to the underlying grid, using transmission images. Contracting HL-1 cells do not express HCN4 (B, C). Large cell clusters that are immunolabeled for HCN4 appear to be located in the vicinity of some contractile areas. Arrowheads indicate some cells specifically stained for HCN4 (C). Scale bars specify 20µm.
